# Combining Fourier Transform Mid-Infrared Spectroscopy with Chemometric Methods to Detect Adulterations in Milk Powder

**DOI:** 10.3390/s19132934

**Published:** 2019-07-03

**Authors:** Lei Feng, Susu Zhu, Shuangshuang Chen, Yidan Bao, Yong He

**Affiliations:** 1 College of Biosystems Engineering and Food Science, Zhejiang University, Hangzhou 310058, China; 2 Key Laboratory of Spectroscopy Sensing, Ministry of Agriculture and Rural Affairs, Hangzhou 310058, China

**Keywords:** milk powder, rice flour, soybean flour, adulteration detection, mid-infrared spectroscopy

## Abstract

Adulteration is one of the major concerns among all the quality problems of milk powder. Soybean flour and rice flour are harmless adulterations in the milk powder. In this study, mid-infrared spectroscopy was used to detect the milk powder adulterated with rice flour or soybean flour and simultaneously determine the adulterations content. Partial least squares (PLS), support vector machine (SVM) and extreme learning machine (ELM) were used to establish classification and regression models using full spectra and optimal wavenumbers. ELM models using the optimal wavenumbers selected by principal component analysis (PCA) loadings obtained good results with all the sensitivity and specificity over 90%. Regression models using the full spectra and the optimal wavenumbers selected by successive projections algorithm (SPA) obtained good results, with coefficient of determination (R^2^) of calibration and prediction all over 0.9 and the predictive residual deviation (RPD) over 3. The classification results of ELM models and the determination results of adulterations content indicated that the mid-infrared spectroscopy was an effective technique to detect the rice flour and soybean flour adulteration in the milk powder. This study would help to apply mid-infrared spectroscopy to the detection of adulterations such as rice flour and soybean flour in real-world conditions.

## 1. Introduction

In general, milk is an important source of protein for adults and children. Specifically, milk is used as an indispensable food in a large number of families to provide daily nutrition for infants. Therefore, the quality and safety of dairy products has been a global concern. There is great demand for high quality milk, and adding simple compounds into milk is a common practice to enhance the nutritional value of milk and increase the economic profits. However, not all additions are legal. After the event of melamine contamination in Chinese infant milk products in 2008, strict rules for milk quality and safety inspection have been developed. However, the milk adulteration still cannot be avoided due to the temptation of achieving high economic profits with lower cost.

Rice flour and soybean flour are harmless and the low prices of these flours make them attractive as potential adulterants in dairy products. Rice flour could be added to milk to increase the solids-not-fat content and viscosity [1,2]. Soybean flour contains plant proteins, thus it also could be used as milk-like products blended in the milk powder to maintain the protein content of the product [3]. These two kinds of flour adulterations will reduce the nutritional value of milk and also have negative effects on those who consume milk as a daily nutritional supplement.

The similarity of milk powder, rice flour and soybean flour makes it difficult to identify the adulterations by the naked eye. Detection of rice flour and soybean flour adulterations is of great importance for milk quality and safety inspection. There are various laboratory-based chemical methods to identify adulterations in milk, but most of them are complex to operate, expensive, reagent wasting, and cannot be used for rapid and accurate large-scale batch detection [4]. Therefore, a rapid, cheap and high-accuracy technique for milk adulteration detection is of great importance.

Mid-infrared spectroscopy is a widely used spectroscopic technique based on the interaction of molecules with electromagnetic radiation in the mid-infrared region. Mid-infrared spectroscopy is capable of exploring the fundamental vibrations and associated rotational-vibrational structure of chemical bonds. Mid-infrared spectroscopy has been implemented as a rapid, accurate, and cheap technique in the detection of milk quality. Cecchinato et al. (2009) predicted rennet coagulation time and curd firmness of milk using mid-infrared spectroscopy. The result indicated that mid-infrared spectroscopy could be implemented for quality detection of milk from different dairy cattle populations [5]. Ye et al. (2016) used mid-infrared spectroscopy to study the variation of the secondary structure of protein induced by temperature in milk powders [6]. Fleming et al. (2017) predicted milk fatty acid content in Canadian dairy cattle with mid-infrared spectroscopy combined with partial least squares (PLS) models. The high accuracies obtained by PLS models demonstrated the possibility to monitor fatty acid contents for cows in milk powder using mid-infrared spectroscopy [7].

Mid-infrared spectroscopy also has been used to identify adulterations in milk. Gondim et al. (2017) proposed a sequential strategy to detect adulterants including formaldehyde, hydrogen peroxide, chloride and so forth in milk using a mid-infrared spectroscopy. Seven different compound adulterations were detected successfully, and 80% of samples were properly classified [8]. He et al. (2010) established two-dimensional (2D) correlation spectroscopy using Fourier transform infrared spectroscopy (FTIR) for the discriminative analysis of melamine, urea, tetracycline and glucose adulterants in milk in milk [9]. Santos et al. (2013) determined the level of adulteration with whey, synthetic milk, synthetic urine, urea and hydrogen peroxide in milk successfully with mid-infrared microspectroscopy [10]. The studies mentioned above mainly focused on the detection of several chemical components.

The objective of this study was to explore the feasibility of using mid-infrared spectroscopy to detect milk adulterated with rice flour and soybean flour. The specific objectives were to: (1) differentiate milk powder, rice flour, soybean flour, milk powder adulterated with rice flour and milk powder adulterated with soybean flour; (2) detect adulterations content in adulterated milk powder quantitatively.

## 2. Materials and Methods

### 2.1. Sample Preparation

Commercial milk powder (Whole milk powder, Anchor, New Zealand), rice flour (Pure rice flour, Jinhuafang, Jinan Province, China) and soybean flour (Pure soybean flour, Wugukang, Shandong Province, China) were purchased online (JingDong Mall). The content of main components in milk powder, rice flour and soybean flour are shown in Table 1.

The milk powders were mixed with the rice flour and the soybean flour respectively. The percentage (by weight) of adulterations were 0%, 5%, 10%, 15%, 20%, 25% and 30%, respectively. For each adulteration percentage, a total of 10 g powders were prepared. Before mixing, the potassium bromide (KBr) powders were dried at 105 °C and placed under an infrared lamp to reduce the influence of water. The mixtures were accurately mixed by grinding manually for 3 min. To obtain samples for mid-infrared spectra acquisition, 0.02 g mixtures of each adulteration percentage were weighed and then mixed with 0.98 g (KBr) powders, and 0.1 g mixtures were weighed and pressed into pellets using a tablet machine. To evaluate the differences among the milk powders, the rice flour and the soybean flour, 120 samples of each powder were prepared, respectively. For milk powder adulterated with rice flour (5%, 10%, 15%, 20%, 25% and 30%) and soybean flour (5%, 10%, 15%, 20%, 25% and 30%), 30 pellets of each adulteration percentage were prepared, respectively. Due to the software error during spectra acquisition, spectra of some samples were missing. 119 samples of each powder without adulterant were obtained. There were 29 samples for milk adulterated with different content of rice flour (5%, 25% and 30%), and there were 29 samples for milk adulterated with different content of soybean flour (10%, 25% and 30%). In total, 177 samples of milk powder adulterated with rice flour (5–30%) and soybean flour (5–30%) were obtained, respectively.

In order to explore whether mid-infrared spectroscopy could be used to distinguish different types of powder, samples of milk powder, rice flour, soybean flour, milk powder adulterated with rice flour and milk powder adulterated with soybean flour were randomly divided into the calibration set and the prediction set at the ratio of 2:1. To detect the content of adulterants in milk powder quantitatively, samples of milk powder adulterated with rice flour (0%, 5%, 10%, 15%, 20%, 25% and 30%) and milk powder adulterated with soybean flour (0%, 5%, 10%, 15%, 20%, 25% and 30%) were also divided into the calibration and the prediction sets at the ratio of 2:1 randomly.

### 2.2. Mid-Infrared Spectra Acquisition

The mid-infrared spectra of samples were acquired by a Fourier Transform Infrared (FTIR) spectrometer (Nicolet iS10, Thermo Scientific, MA, USA) in the spectral range of 400–4000 cm^−1^. The resolution was set as 4 cm^−1^. The spectra acquisition mode was transmittance. The scanning time of each sample was set as 32, and the average spectrum of the 32 scans was used as the spectrum of the sample.

### 2.3. Chemometric Methods

#### 2.3.1. Principal Component Analysis

Principal component analysis (PCA) is a widely used feature extraction and data dimension reduction method. PCA linearly transforms the original data into new orthogonal variables (called principal component, PC), and the PCs are ranked from high to low according to the explained variances. In general, the first few PCs explained the most of the total variance. In this study, PCA was used to form the scores scatter plot and identify the optimal wavenumbers by loadings inspection [11,12,13,14].

#### 2.3.2. Calibration Models

In this study, calibration models including discriminant models and regression models were used. Discriminant models were built to discriminate the milk powders, rice flour and soybean flour and to discriminate the milk powder adulterated with rice flour and soybean flour. Regression models were used to quantitatively detect adulteration content. Partial least squares (PLS), extreme learning machine (ELM) and support vector machine (SVM) were all used to build both kinds of models.

PLS is the mostly used chemometric method in spectral data analysis. PLS reveals the linear relationship between the independent matrix (X) and the dependent variables (Y). PLS transforms X and Y into new variables at the same time. PLS tries to maximize the variances of the new variables and find the maximum correlation between the new variables of X and Y. For regression and discrimination, the modelling procedures are the same. For regression, Y is the real numbers representing the features to be predicted. For discrimination, Y is the integer numbers or dummy numbers representing the categories [15,16,17,18]. To conduct PLS, the optimal number of latent variables were determined by leave-one-out cross validation. ELM is a widely used feedforward neural network. ELM chooses the weights connecting inputs to hidden nodes randomly without modification. The main parameter of ELM to be determined is the number of neurons in the hidden layer. ELM has the same training procedure for classification and regression [19,20,21].

ELM has the characteristics of good generalization ability and fast computation. Activation function is the key to ELM models, and there are various activation functions such as Sigmoid function, sine function and radial basis function, etc. In this study, radial basis function (RBF) was used as activation function. To conduct ELM, the optimal number of neurons in the hidden layer was determined by comparing the performances of ELM models using different number of neurons, and the ELM models were also built by using leave-one-out cross validation.

SVM is a machine learning method for classification and regression. SVM tries to map the original data into a high-dimension space and build hyperplanes in the high-dimension space to maximally separate samples for classification. For regression, SVM tries to map the original data into a high-dimension space and solve the linear regression problem in the high-dimension space. Kernel function is the key to the mapping. The generally used kernel functions are RBF, polynomial, linear and Sigmoid kernel functions. In this study, RBF was used as kernel function for both classification and regression [22,23]. To conduct SVM, the optimal parameters C and g were determined by a grid-search procedure from 2^−8^ to 2^8^, and the SVM models were built using five-fold cross validation.

### 2.4. Variable Selection

The acquired mid-infrared spectra contained background information and redundant information. Optimal wavenumbers selection is used to select a few most informative wavenumbers for further analysis to reduce the influence of background information and redundant information. With fewer wavenumbers, the model inputs will be reduced and the model will be simplified. In this study, optimal wavenumbers were selected for discrimination of milk and its adulterations (rice flour and soybean flour), discrimination of milk adulterated with different adulterations and determination of the adulteration content.

Loadings of variables are the correlation coefficient between the original variables and new variables in each PC of PCA. In each PC, absolute loading value indicates the importance of the corresponding variable. Loadings of the first few PCs are therefore used for variable selection. In this study, PCA loadings were used to select optimal wavenumbers for discrimination of milk and its adulterations (rice flour and soybean flour), discrimination of milk adulterated with different adulterations.

Successive projections algorithm (SPA) is a forward variable selection method. SPA is conducted in two steps. Firstly, SPA calculates the projections of one variable on the others and selects the variables with maximum projection into the candidate subset. The variables are ranked according to the projections. Then a calibration model is built on the different number of variables to evaluate the selected variables. The variables with the best model performances are selected [24,25,26,27,28]. In this study, multiple linear regression was used to evaluate the selected variables for content determination.

### 2.5. Software and Model Evaluation

In this study, PLS and second derivative were conducted on Unscrambler^®^ 10.1 (CAMO AS, Oslo, Norway), while PCA, ELM, and SVM were performed on MATLAB R 2014b (The Math Works, Natick, MA, USA).

Receiver operating characteristics (ROC) is used to illustrate the discriminant performances of models. True Positive (TP), False Positive (FP), True Negative (TN) and False Negative (FN) are terminologies used in the description of ROC [29]. The equations of sensitivity and specificity are presented as follows:

(1) $$Sensitivity=\frac{TP}{TP+FN}\times 100\%$$

(2) $$Specificity=\frac{TN}{FP+TN}\times 100\%$$

where *TP* means the classified result and the actual label are both positive. *FP* stands for the classified result is positive while the actual label is negative. *TN* means the classified result and the actual label are both negative. *FN* means the classified result is negative while the actual label is positive. For a good classifier, sensitivity and specificity should be close to 100%.

The performances of regression models were evaluated by the coefficient of determination (R^2^), root mean square error of the calibration set (RMSEC) and prediction set (RMSEP). The residual predictive deviation (RPD) was also used to evaluate the model performances. According to Zornoza et al. (2008), models with R^2^ over 0.9 or RPD over 3 showed excellent performances, models with R^2^ between 0.81 and 0.90 or RPD between 2.5 and 3 showed good performances and R^2^ between 0.61 and 0.8 or RPD between 2.0 and 2.5 indicated that the models could be used for prediction [30].

## 3. Results and Discussion

### 3.1. Spectra Profiles

**Figure 1:**
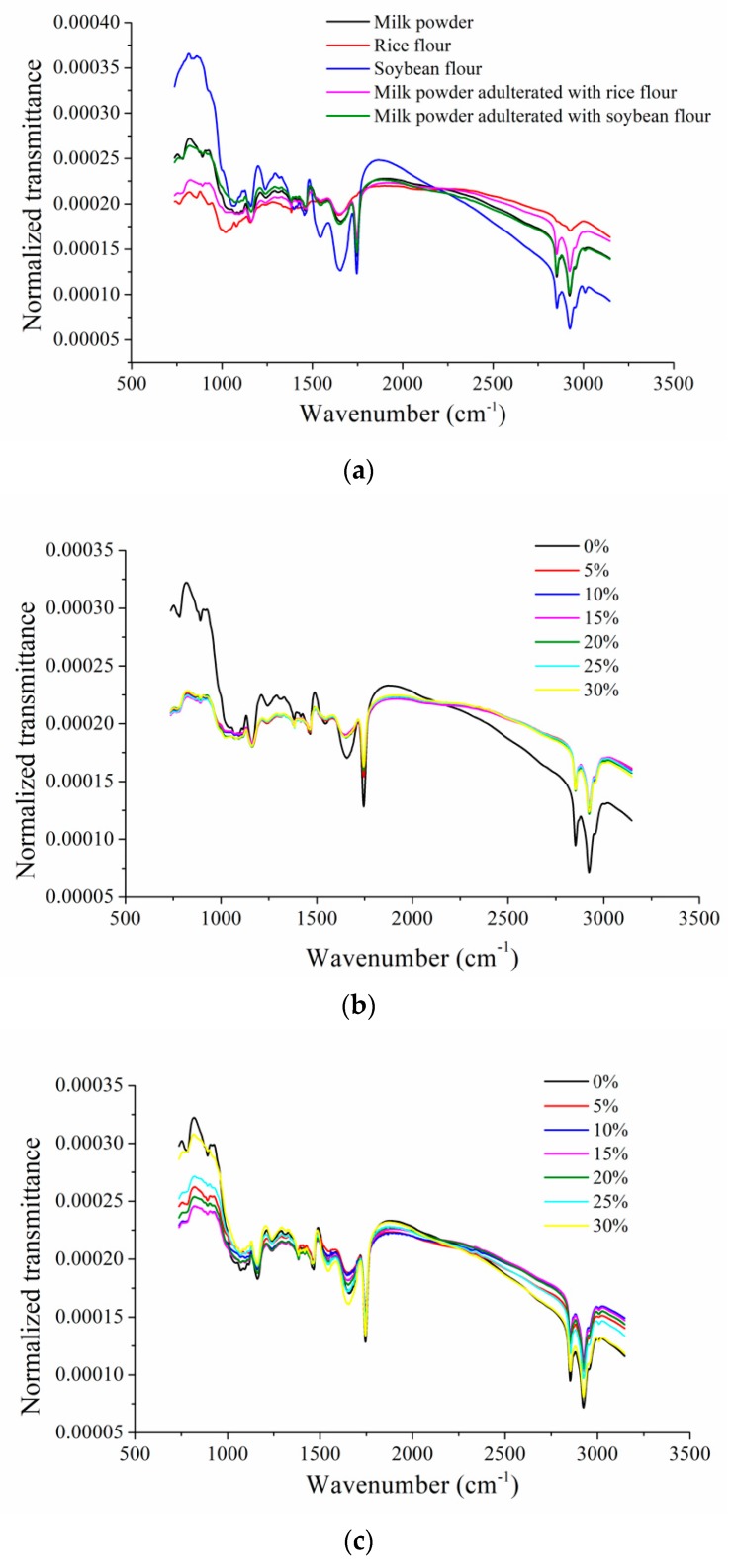
Average spectra of samples: (**a**) Average spectra of milk powder, rice flour, soybean flour, milk powder adulterated with rice flour and milk powder adulterated with soybean flour; (**b**) Average spectra with of milk powder adulterated with different content of rice flour (0%, 5%, 10%, 15%, 20%, 25% and 30%); (**c**) Average spectra of milk powder adulterated with different content of soybean flour (0%, 5%, 10%, 15%, 20%, 25% and 30%).

There was obvious noise in the head and end of the spectra due to system error. Therefore, only the spectra at the range of 737.1565 cm^−1^–3147.2580 cm^−1^ were analyzed in this study. The mid-infrared spectra were preprocessed by wavelet transform to reduce the noise, and the wavelet function was Daubechies 8 with the decomposition level being 3. The denoised spectra were then preprocessed by area normalization to reduce the light variation among different samples. Figure 1a shows the average spectra of milk powder, rice flour, soybean flour, milk powder adulterated with rice flour and milk powder adulterated with soybean flour. To match the sample number of adulterated samples, 30 milk powder samples were randomly selected. Differences could be observed among different samples. The spectral peaks and valleys in the spectra were quite similar. Although the spectral curves of different samples were similar, their transmittance value were different, and some of the wavenumbers showed obvious differences. The spectral differences of milk powder adulterated with different contents were also observed.

As shown in Figure 1a, differences of transmittance spectra could be found among the average spectrum of milk powder, rice flour and soybean flour. Specifically, the two transmittance valleys near 2800 cm^−1^ are characteristics of CH_2_ symmetric stretching of fatty acid [31]. The valley near 1700 cm^−1^ is associated with amide I and II, which can be connected with protein content [32]. Two characteristics valleys around 1400 and 1600 cm^−1^ are due to symmetric and asymmetric stretching of C-O bonds from carboxylate groups [33]. The peak in the range of 700–1200 cm^−1^ is assigned to carbohydrate chain vibrations [34]. The differences of these wavebands reflected the differences of the related compositions differences of fat, protein and carbohydrate. The spectral differences matched with the differences on the content of fat, protein and carbohydrate in Table 1. However, these spectral differences presented the differences of compositions qualitatively, and the quantitative relationship between the spectral differences and composition differences needs further exploration. The characteristic bands in this region can be used for adulterants detection.

Figure 1b,c shows the average spectra of milk powder adulterated with different contents of rice flour (0%, 5%, 10%, 15%, 20%, 25% and 30%) and different contents of soybean flour (0%, 5%, 10%, 15%, 20%, 25% and 30%), respectively. The wavebands with differences in Figure 1b,c were similar to those in Figure 1a. The milk powder adulterated with different contents of rice flour and soybean flour varied in the chemical compositions (fat, protein and carbohydrate, etc.), which resulted in the differences in transmittances spectra. For milk powder adulterated with rice flour, larger differences could be found between the unadulterated milk powder and the adulterated one, and the differences among adulterated samples were smaller. For milk powder adulterated with soybean flour, differences could be found among samples adulterated with different content of soybean flour (0%, 5%, 10%, 15%, 20%, 25% and 30%), but the differences were small.

### 3.2. PCA Scores Scatter Plot Analysis

**Figure 2:**
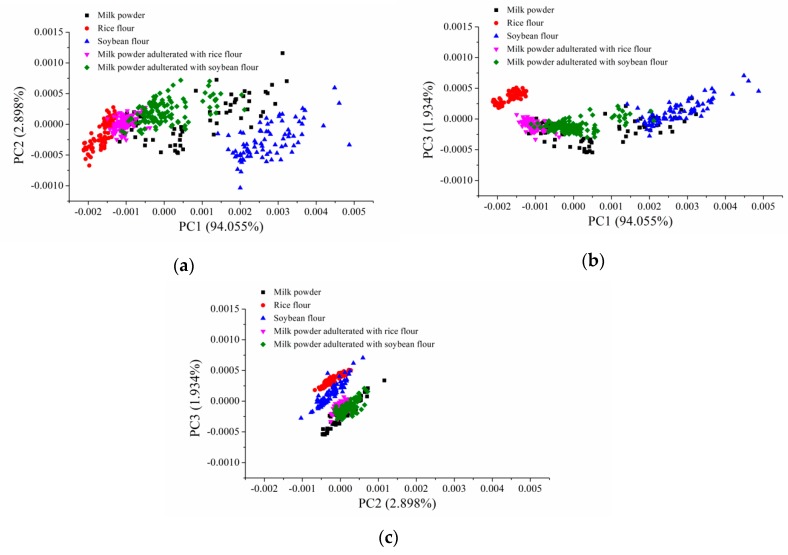
Scores scatter plots: (**a**) PC1 vs PC2; (**b**) PC1 vs PC3; (**c**) PC2 vs PC3. (PC means principal component).

PCA was conducted to explore the sample distribution in the scores scatter plots. PCA was conducted on the calibration set, and PCA scores scatter plots of PC1 vs PC2, PC1 vs PC3 and PC2 vs PC3 are shown in Figure 2, the first three PCs contributed to 98.888% of the total variances. Samples of milk powder, rice flour, soybean flour, milk powder adulterated with rice flour and milk powder adulterated with soybean flour showed clear clusters in different scores scatter spaces, and clear separation could be observed. Moreover, overlaps could be found among different kinds of flour. PCA scores scatter plots indicated that there were differences among milk powder, rice flour, soybean flour, milk powder adulterated with rice flour and milk powder adulterated with soybean flour. There were potentials to identify these samples using mid-infrared spectroscopy.

### 3.3. Classification Models of Powder Samples

#### 3.3.1. Classification Models Using Full Spectra

Identification of milk powder, rice flour, soybean flour, milk powder adulterated with rice flour and milk powder adulterated with soybean flour provided the first screen of adulterated milk. The category values of milk powder, rice flour, soybean flour, milk powder adulterated with rice flour and milk powder adulterated with soybean flour were coded as 00001, 00010, 00100, 01000, 10000 for PLS models, and assigned as 1, 2, 3, 4 and 5 for SVM models and ELM models. The samples were split into the calibration set and the prediction set at the ratio of 2:1. There were 480 samples in the calibration set and 231 samples in the prediction set.

The discriminant results are shown in Table 2. Good classification results were obtained, with most of the sensitivity and specificity over 90%, although performances of different models varied. The prediction results of the first category (milk) seemed to be easily confused with the fourth category (milk adulterated with rice flour) for PLS and SVM models, with sensitivity in the range of 60%–75%, which could be caused by the similarity of their spectral curves. ELM model outperformed the other two models, with sensitivity and specificity of calibration set all reaching over 99%, and the two indicators of prediction set reaching 87%, 97%, respectively. The good results of the ELM model indicated that it was effective to classify milk powder, rice flour, soybean flour, milk powder adulterated with rice flour and milk powder adulterated with soybean flour by the mid-infrared spectroscopy.

#### 3.3.2. Optimal Wavenumbers Selection

**Figure 3:**
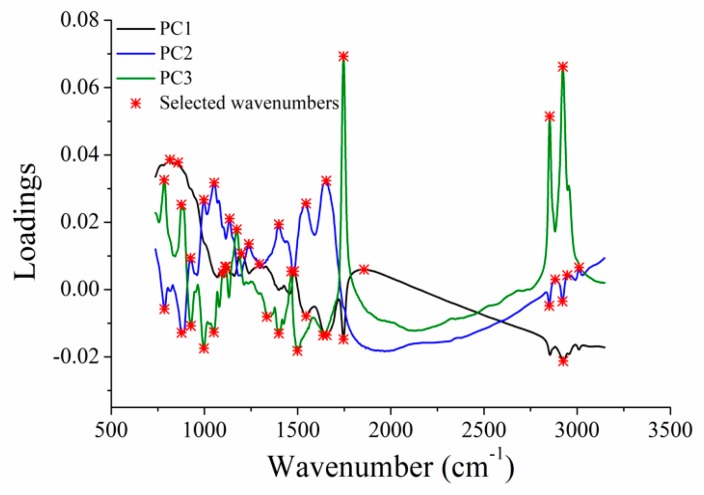
Optimal wavenumbers selected by loadings of PC1, PC2 and PC3.

There were 5000 variables in each spectra, and some of the variables were uninformative. These uninformative variables might affect the modelling performances. Therefore, it was necessary to select informative variables contributing more to the classification to reduce the number of input variables, which could simplify the models and improve the robustness of models. As shown in Figure 3, loadings of the first three PCs obtained by PCA were used to select informative variables. The first three PCs explained over 98% of the total variances, and scores scatter plots of the first three PCs showed clear separation of different kinds of samples. Table 3 shows the selected optimal wavenumbers, and 42 wavenumbers were selected for classification.

#### 3.3.3. Classification Models Using Optimal Wavenumbers

PLS, SVM and ELM models were also built using the selected optimal wavenumbers. The results of the classification models are presented in Table 4. Good classification results were obtained with most of the sensitivity and sensitivity indices over 90% for three models. As the same as the results of models using full spectra, the prediction results of the first category (milk) were easily confused with the fourth category (milk adulterated with rice flour) for PLS and SVM models. ELM model using optimal wavenumbers still achieved better performance, with sensitivity and specificity of calibration set all reaching over 99%, and the same indicators of prediction set reaching 92%, 98%, respectively. The good results of three models using selected wavenumbers indicated that it was feasible to identify different powder samples with the selected optimal wavenumbers instead of full spectra.

The performances of models using full spectra and optimal wavenumbers were close. The number of input variables of these models reduced 99.16% by using the optimal wavenumbers. With the significant reduction of the number of wavenumber, the close results indicated the effectiveness of optimal wavenumbers selection. Moreover, different adulteration types with different adulteration content were accurately identified in all models. For models using full spectra and the selected optimal wavenumbers, ELM models outperformed the other two models, which indicated that in this study, ELM was more preferable. These results showed the feasibility of using mid-infrared spectroscopy to firstly screen milk, adulterations and milk with adulterations.

### 3.4. Determination of Adulterations Content in the Adulterated Milk

#### 3.4.1. Calibration Models Using Full Spectra

It was noted that the number of samples with each adulteration degree was 29 or 30. To build calibration models for determination of adulterations content in milk, 30 samples of pure milk powder, 30 samples of pure rice flour and 30 samples of pure soybean flour were randomly selected. The samples were randomly divided into the calibration set and the prediction set at the ratio of 2:1, and there were 140 samples in the calibration set and 67 samples in the prediction set. Performances of PLS, SVM and ELM models using full spectra for rice flour adulteration and soybean flour adulteration are presented in Table 5. The procedure to determine model parameters for regression was the same as that for classification.

The calibration models all obtained good performances, with R^2^_c_ and R^2^_p_ over 0.9 and RPD over 3. Detection results of soybean flour adulteration were slightly better than those of rice flour adulteration. ELM models obtained the best performances for both kinds of adulteration. PLS models outperformed the SVM models. ELM model for soybean flour adulteration content detection showed the best performances with RPD over 8. The good prediction results of all the three models indicated that mid-infrared spectroscopy was an efficient technique to determine adulteration (rice flour and soybean flour) content in milk powder.

#### 3.4.2. Optimal Wavenumbers Selection

To simplify the models, SPA was used to select optimal wavenumbers. The number of wavenumbers to be selected was limited to 10–50. The selected wavenumbers are shown in Table 6. Different numbers of optimal wavenumbers were selected, and the selected wavenumbers were merely the same. The differences of selected wavenumbers indicated the chemical composition differences between milk powder adulterated with rice flour and milk powder adulterated with soybean flour.

#### 3.4.3. Calibration Models Using Optimal Wavenumbers

**Figure 4:**
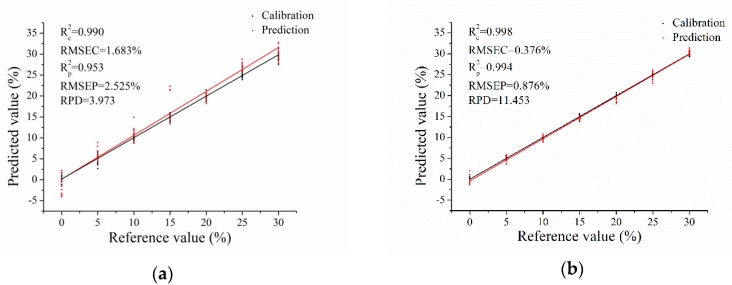
The plots of the prediction value versus the reference value of ELM models using the selected optimal wavenumbers for (**a**) Milk adulterated with rice flour; (**b**) Milk adulterated with soybean flour.

PLS, SVM and ELM models were established using the selected optimal wavenumbers. The results are shown in Table 7. All models obtained good performances, with R^2^_c_ and R^2^_p_ over 0.9 and RPD over 3, indicating excellent performances. Detection results of soybean flour adulteration were slightly better than those of rice flour adulteration. The results obtained by ELM models were slightly better than PLS and SVM models. ELM models performed the best for soybean flour adulteration content detection, with R^2^_c_, R^2^_p_ and RPD being 0.998, 0.994 and 11.453, respectively. The plots of the prediction value versus the reference value of ELM models using the selected optimal wavenumbers for the two adulterations are presented in Figure 4. It could be seen that the performances of soybean adulteration content detection were better than those of rice adulteration content detection.

A comparison was made between the models using full spectra and those using the selected optimal wavenumbers. In general, models using the selected optimal wavelengths performed slightly better than those using the full spectra. Furthermore, the number of input variables of the three models reduced 99.56% for rice flour adulteration content determination and 99.34% for soybean adulteration determination. The good results of all models indicated that the selected optimal wavenumbers could be used for milk adulteration content determination. In general, for models using full spectra and the selected optimal wavenumber, ELM model obtained slightly better results than the other two models. These results showed that ELM might be preferable for milk adulteration content determination.

## 4. Conclusions

Mid-infrared spectroscopy was successfully used to identify milk powder, rice flour, soybean flour, milk powder adulterated with rice flour and milk powder adulterated with soybean flour (classification), then mid-infrared spectroscopy was used to determine adulteration (rice flour and soybean flour) content in milk (regression). PLS, SVM and ELM models were used as classification and regression methods. ELM models using full spectra and optimal wavenumbers obtained decent performances of classification. The sensitivity and specificity of both calibration set and the prediction set for identifying milk powder, rice flour, soybean flour, milk powder adulterated with rice flour and milk powder adulterated with soybean flour were over 87%. The R^2^_c_ and R^2^_p_ of all models’ adulteration content determination was over 0.9, and the corresponding RPD was all over 3. These good performances indicated that it was feasible to detect milk adulterations and the adulterations content for rice flour adulteration and soybean flour adulteration by mid-infrared spectroscopy. In future works, more adulterations content, including the lower content of adulterations, will be explored for better and more robust models. Moreover, the results will help to conduct real-world detection for milk-like adulterations (rice flour, soybean flour, etc.) in milk powder.

## Figures and Tables

**Table 1:** The content of main components in milk powder, rice flour and soybean flour.

Component	Content (g/100g)		
	Milk Powder	Rice Flour	Soybean Flour
Protein	24	0.3	32.8
Fat	28.8	0.3	18.3
Carbohydrate	38.4	95.6	30.5

**Table 2:** Confusion matrix of models using full spectra.

Model	Par. ^1^		Sample Number	Pre. ^2^					Sensitivity (%)	Specificity (%)
				M.^3^	R.	S.	MR.	MS.		
PLS	17	Cal. ^4^	M. (80)	80	0	0	0	0	100.00	100.00
			R. (80)	0	80	0	0	0	100.00	100.00
			S. (80)	0	0	80	0	0	100.00	100.00
			MR. (120)	0	0	0	120	0	100.00	99.72
			MS. (120)	0	0	0	1	119	99.17	100.00
		Pre.	M. (39)	27	0	0	12	0	69.23	100.00
			R. (39)	0	39	0	0	0	100.00	100.00
			S. (39)	0	0	39	0	0	100.00	100.00
			MR. (57)	0	0	0	57	0	100.00	93.10
			MS. (57)	0	0	0	0	57	100.00	100.00
SVM	147, 0.0039	Cal.	M. (80)	80	0	0	0	0	100.00	100.00
			R. (80)	0	80	0	0	0	100.00	100.00
			S. (80)	0	0	80	0	0	100.00	100.00
			MR. (120)	0	0	0	120	0	100.00	99.72
			MS. (120)	0	0	0	1	119	99.17	100.00
		Pre.	M. (39)	29	0	0	10	0	74.36	98.44
			R. (39)	0	39	0	0	0	100.00	100.00
			S. (39)	0	0	39	0	0	100.00	100.00
			MR. (57)	0	0	0	57	0	100.00	94.15
			MS. (57)	3	0	0	0	54	94.74	100.00
ELM	78	Cal.	M. (80)	80	0	0	0	0	100.00	100.00
			R. (80)	0	80	0	0	0	100.00	100.00
			S. (80)	0	0	80	0	0	100.00	100.00
			MR. (120)	0	0	0	120	0	100.00	99.72
			MS. (120)	0	0	0	1	119	99.17	100.00
		Pre.	M. (39)	34	0	0	5	0	87.18	99.48
			R. (39)	0	39	0	0	0	100.00	100.00
			S. (39)	0	0	39	0	0	100.00	100.00
			MR. (57)	1	0	0	56	0	98.25	97.13
			MS. (57)	0	0	0	0	57	100.00	100.00

^1^ Parameter, the parameter of the partial least squares (PLS) model is the optimal number of latent variables, the parameter of the support vector machine (SVM) model is the penalty coefficient C and radial basis function (RBF) kernel parameter g, the parameter of the extreme learning machine (ELM)model is number of the hidden layer neurons; ^2^ Prediction set; ^3^ M., R., S., MR. and MS. are assigned respectively as milk powder, rice flour, soybean flour, milk powder adulterated with different contents of rice flour and milk powder adulterated with different contents of soybean flour; ^4^ Calibration set.

**Table 3:** Optimal wavenumbers selected by PCA loadings.

Methods	Number	Wavenumbers (cm^−1^)
PCA loadings	42	784, 786, 815, 859, 877, 877, 926, 928, 997, 997, 1051,1052,
		1098, 1111, 1116, 1134, 1173, 1198, 1238, 1294, 1334, 1399,
		1400, 1467, 1481, 1499, 1545, 1546, 1637, 1654, 1656, 1746,
		1746, 1856, 2850, 2852, 2882, 2922, 2923, 2926, 2947, 3010

**Table 4:** Confusion matrix of models using optimal wavenumbers.

Model	Par. ^1^		Sample Number	Pre. ^2^					Sensitivity (%)	Specificity (%)
				M.^3^	R.	S.	MR.	MS.		
PLS	12	Cal. ^4^	M. (80)	78	0	0	2	0	97.50	100.00
			R. (80)	0	80	0	0	0	100.00	100.00
			S. (80)	0	0	80	0	0	100.00	100.00
			MR. (120)	0	0	0	117	3	97.50	98.89
			MS. (120)	0	0	0	2	118	98.33	99.16
		Pre.	M. (39)	21	0	0	18	0	53.85	100.00
			R. (39)	0	38	0	1	0	97.44	100.00
			S. (39)	0	0	37	0	2	94.87	100.00
			MR. (57)	0	0	0	55	2	96.49	88.95
			MS. (57)	0	0	0	0	57	100.00	97.42
SVM	256, 0.5743	Cal.	M. (80)	80	0	0	0	0	100.00	100.00
			R. (80)	0	80	0	0	0	100.00	100.00
			S. (80)	0	0	80	0	0	100.00	100.00
			MR. (120)	0	0	0	120	0	100.00	99.72
			MS. (120)	0	0	0	1	119	99.17	100.00
		Pre.	M. (39)	24	0	0	15	0	61.54	97.92
			R. (39)	0	39	0	0	0	100.00	100.00
			S. (39)	0	0	39	0	0	100.00	100.00
			MR. (57)	0	0	0	57	0	100.00	91.18
			MS. (57)	4	0	0	0	53	92.98	100.00
ELM	218	Cal.	M. (80)	80	0	0	0	0	100.00	100.00
			R. (80)	0	80	0	0	0	100.00	100.00
			S. (80)	0	0	80	0	0	100.00	100.00
			MR. (120)	0	0	0	120	0	100.00	99.72
			MS. (120)	0	0	0	1	119	99.17	100.00
		Pre.	M. (39)	36	0	0	3	0	92.31	100.00
			R. (39)	0	39	0	0	0	100.00	100.00
			S. (39)	0	0	39	0	0	100.00	100.00
			MR. (57)	0	0	0	57	0	100.00	98.28
			MS. (57)	0	0	0	0	57	100.00	100.00

^1.^ Parameter, the parameter of the partial least squares (PLS) model is the optimal number of latent variables, the parameter of the support vector machine (SVM) model is the penalty coefficient C and radial basis function (RBF) kernel parameter g, the parameter of the extreme learning machine (ELM) model is number of the hidden layer neurons; ^2^ Prediction set; ^3^ M., R., S., MR. and MS. are assigned respectively as milk powder, rice flour, soybean flour, milk powder adulterated with different contents of rice flour and milk powder adulterated with different contents of soybean flour; ^4^ Calibration set.

**Table 5:** Results of determination of adulterations content in milk using full spectra.

Adulterations	Model	Par. ^1^	R^2^_c_^2^	RMSEC ^3^	R^2^_p_^4^	RMSEP ^5^	RPD ^6^
Rice Flour	PLS	7	0.969	1.772	0.945	2.719	3.690
	SVM	64, 0.0055	0.997	0.592	0.915	3.060	3.279
	ELM	37	0.986	1.156	0.949	2.424	4.139
Soybean Flour	PLS	3	0.945	2.335	0.953	2.350	4.269
	SVM	16, 0.0039	0.999	0.394	0.939	2.523	3.977
	ELM	79	0.998	0.440	0.988	1.129	8.887
	ELM	79	0.998	0.440	0.988	1.129	8.887

^1^ Parameter; ^2^ Coefficient of determination of calibration set; ^3^ Root mean square error of the calibration set; ^4^ Coefficient of determination of prediction set; ^5^ Root mean square error of the prediction set; ^6^ Residual predictive deviation, the value of the standard deviation of prediction for calculation of RPD was 10.033.

**Table 6:** Optimal wavenumbers selected by PCA loadings.

Adulterations	Methods	Number	Wavenumbers (cm^−1^)
Rice Flour	SPA	22	743, 767, 759, 821, 845, 879, 895, 922, 964, 1022, 1068, 1145, 1176, 1217, 1462, 1507, 1653, 1622, 1748, 2846, 2966, 3147
Soybean Flour	SPA	33	744, 752, 761, 768, 794, 802, 809, 836, 852, 860, 867, 888, 903, 929, 933, 995, 1030, 1068, 1137, 1190, 1465, 1507, 1538, 1560,1615, 1644, 1704, 1734, 1739, 1748, 2839, 3010, 3147

**Table 7:** Results of determination of adulterations content in milk using optimal wavenumbers.

Adulterations	Model	Par. ^1^	R^2^_c_^2^	RMSEC ^3^	R^2^_p_^4^	RMSEP^5^	RPD ^6^
Rice Flour	PLS	7	0.972	1.683	0.945	2.514	3.991
	SVM	256, 0.2500	0.984	1.252	0.939	2.577	3.893
	ELM	28	0.990	1.009	0.953	2.525	3.973
Soybean Flour	PLS	3	0.945	2.337	0.951	2.366	4.240
	SVM	32, 0.1768	0.996	0.633	0.958	2.117	4.739
	ELM	60	0.998	0.376	0.994	0.876	11.453

^1^ Parameter; ^2^ Coefficient of determination of calibration set; ^3^ Root mean square error of the calibration set; ^4^ Coefficient of determination of prediction set; ^5^ Root mean square error of the prediction set; ^6^ Residual predictive deviation, the value of the standard deviation of prediction for calculation of RPD was 10.033.
